# Discovery of novel anti-tumor compounds targeting PARP-1 with induction of autophagy through *in silico* and *in vitro* screening

**DOI:** 10.3389/fphar.2022.1026306

**Published:** 2022-10-24

**Authors:** Danfeng Shi, Qianqian Pang, Qianyu Qin, Xinsheng Yao, Xiaojun Yao, Yang Yu

**Affiliations:** ^1^ Guangdong Province Key Laboratory of Pharmacodynamic Constituents of TCM and New Drugs Research, International Cooperative Laboratory of Traditional Chinese Medicine Modernization and Innovative Drug Development of Ministry of Education (MOE) of China, Institute of Traditional Chinese Medicine and Natural Products, College of Pharmacy, Jinan University, Guangzhou, China; ^2^ State Key Laboratory of Quality Research in Chinese Medicine, Macau Institute for Applied Research in Medicine and Health, Macau University of Science and Technology, Taipa, Macau, China

**Keywords:** PARP-1 inhibitor, anti-tumor, *in silico* screening, autophagy, allosteric

## Abstract

Poly (ADP-ribose) polymerase 1 (PARP-1) is a critical enzyme involved in DNA damage repair and recombination, and shows great potential for drug development in the treatment of cancers with defective DNA repair. The anti-tumor activities of PARP-1 inhibitors are regulated by both inhibition activities and allosteric mechanisms of PARP-1, and may also be involved in an autophagy-mediated process. Screening PARP-1 inhibitors with potential allosteric mechanisms and induced autophagy process could achieve elevated potency toward cancer cell killing. In this study, we tried to discover novel anti-tumor compounds targeting PARP-1 by computer simulations and *in vitro* screening. In order to filter PARP-1 inhibitors that could affect the folding state of the helix domain (HD) on PARP-1, the free energy contribution of key residues on HD were systematically analyzed using the ligand-binding crystal structures and integrated into *in silico* screening workflow for the selection of 20 pick-up compounds. Four compounds (Chemdiv codes: 8012-0567, 8018-6529, 8018-7168, 8018-7603) were proved with above 40% inhibitory ratio targeting PARP-1 under 20 μM, and further performed binding mode prediction and dynamic effect evaluation by molecular dynamics simulation. Further *in vitro* assays showed that compounds 8018-6529 and 8018-7168 could inhibit the growth of the human colorectal cancer cell (HCT-116) with IC50 values of 4.30 and 9.29 μM and were accompanied with an induced autophagy process. Taken together, we discover two novel anti-tumor compounds that target PARP-1 with an induced autophagy process and provide potential hit compounds for the anti-cancer drug development.

## 1 Introduction

Poly (ADP-ribose) polymerase 1 (PARP-1) is a critical enzyme in the cell nucleus that responds to the damage repair of single- and double-stranded DNA breaks (SSBs and DSBs) (Ame et al., 2004). Upon relocating and binding to DNA damage sites, PARP-1 could activate the poly (ADP-ribosyl)ation by transforming ADP-ribose unit from nicotinamide adenine dinucleotide (NAD+) to various substrate proteins or nucleic acids (Langelier et al., 2018a). This post-translational modification (PTM) process on PARP-1 itself or histones in nucleosomes proximal to the break could rebuild the connection between DNA damage and chromatin modification. It has been found that the lacking of both PARP-dependent SSB repair and BRCA-dependent DSB repair is not tolerable for cell survival (Helleday, 2011), which is also known as a phenomenon of synthetic lethality (O’Neil et al., 2017). The PARP-1 inhibitors can selectively kill the cancer cells with homologous recombination (HR) defect caused by BRCA1/2 mutations, and have been approved for the therapy of cancers such as ovarian cancer, prostate cancer and breast cancer, etc (Rouleau et al., 2010; Lin and Kraus, 2017; Lord and Ashworth, 2017). Clinically, these PARP inhibitors have been mainly applied for the maintenance therapy of malignancies or the treatment for recurrent cancer, and the combination therapies with other kind of inhibitors also show great potential for the overcome of tumor resistance (Rose et al., 2020; Tung and Garber, 2022).

PARP-1 inhibitors with distinct scaffolds exhibit vastly different anti-tumor efficacy in the clinic (Shen et al., 2013). Basically, all PARP-1 inhibitors were engaged in a competitive manner to the nicotinamide portion of NAD + by forming hydrogen bonding with the backbone amide of Gly863 and the side chain oxygen of Ser904. The typical scaffold-hopping optimization of benzamide and cyclic lactam scaffolds has generated a variety of active scaffolds with excellent inhibitory activities (Wang et al., 2016). Recently, it is validated that PARP-1 inhibitors could confer its cellular toxicity through two ways simultaneously, inhibiting poly (ADP-ribosyl)ation by the occupancy of the ADP-ribosyl transferase (ART) domain and prolonging the retention of PARP-1 on DNA damage (Langelier et al., 2018b; Zandarashvili et al., 2020). The retention of PARP-1 on DNA damage is dependent on an allosteric regulatory effect which is engaged in the conformational unfolding of the helix domain (HD) adjacent to the ART domain. The mechanistic studies also showed that the cellular toxicities of PARP-1 inhibitors could be elevated with the stronger abilities to destabilize the conformation of HD (Langelier et al., 2018b; Zandarashvili et al., 2020; Rouleau-Turcotte et al., 2022). It is indicated that the inhibitors with contacts with HD could trap PARP-1 on DNA damage and show enhanced killing ability against cancer cells. Therefore, the discovery and design of novel PARP-1 inhibitors can be optimized in view of the effect on the HD.

In addition to the direct targeted regulation effects on DNA repair process, PARP-1 inhibitors also have shown great potential to intervene the autophagy process which was induced by the DNA damage (Zhang et al., 2018) and shows great potential for synergistic therapeutic effect (Munoz-Gomez et al., 2009; Arun et al., 2015; Jiang et al., 2018; Casili et al., 2020). Typically, PARP-1 inhibitors have been reported to induce autophagy in a variety of tumor models, including ovarian cancer (Santiago-O'Farrill et al., 2020), chronic myeloid leukemia (Liu et al., 2019) and hepatocellular carcinoma (Zai et al., 2020), and the cotreatment with an autophagy inhibitor (chloroquine) may further expand the therapeutic efficacy of PARP inhibitors. The induction of autophagy by PARP-1 inhibitors may further provide an opportunity to improve the efficacy against tumors.

Methods like molecular docking and molecular dynamics simulation have provided a series of ways to analyze the interactions between the ligand and binding sites (Liu et al., 2018). It is hypothesized that a systematic analysis of different conformational states of complex will provide valuable information about the ligand binding before the screening process (Cooper et al., 2011; Maveyraud and Mourey, 2020). The different conformational states of domains or residues around the binding site have significant effect on the binding mode of ligands and will further affect the docking-based screening (Chen et al., 2006). Currently, more than 40 crystal structures of PARP-1 complex were reported in the PDB database (Sussman et al., 1998), providing abundant information about binding modes and conformational changes upon ligand binding. By decomposing the total binding affinity to the contribution of every single residue, the key residues around the binding sites could be effectively recognized. In order to discover novel PARP-1 inhibitor, the systematic analysis of the residues around ART domain and the HD were performed before *in silico* screening process. The reveal of the dynamic and energetic characters of PARP-1 complex may improve the performance of *in silico* screening.

In this study, an integrated approach of *in silico* and *in vitro* screening was performed to discover novel PARP-1 inhibitors. In order to enhance the effectiveness of virtual screening and find molecules with potential allosteric effect, the pocket residues on ART domain and HD were analyzed by their effect on the binding free energy with PARP-1 inhibitors. Multiple linear regression was applied to re-weight the contribution of these key residues and applied in the virtual screening. Then, a protocol of virtual screening workflow is evaluated and designed for the discovery of novel scaffolds of PARP-1 inhibitors, and 20 compounds were purchased from the Chemdiv database for *in vitro* assays. The inhibition ratio was evaluated by chemiluminescent PARP Assay Kit assay, and four hit compounds were found at micromolar level. Molecular dynamics simulation combined with binding free energy calculation revealed the binding modes, and further cell experiments also validated the potential anti-tumor effect of these compounds and the process of inducing autophagy. These compounds can provide new scaffolds for developing novel PARP-1 inhibitors applicable to cancer therapy using further hit-to-lead structural modification strategies.

## 2 Materials and methods

### 2.1 Crystal structure collection and binding mode analysis

A series of crystal structures of the PARP-1 in complex with structurally diverse inhibitors were retrieved from the PDB database (https://www.rcsb.org/). The crystal structures with the existence of the HD in crystal structures of complex (Supplementary Table S1) were further selected for binding mode analysis. The crystal structures were prepared in the Protein Preparation Wizard module in Schrödinger Software Suite (Schrödinger, LLC: New York, NY, 2015). The crystalline water molecules and ions in each crystal structure were removed, while the ligand and protein were prepared by adding hydrogens, filling in missing side chains, and assigning the protonation state of residues at pH value of 7.0. Then, the mass centroids of the native ligands in the crystal structures were defined as the centers of the binding pockets. All the ligands were extracted from the crystal structures and prepared using MMFFs force field, with a target pH of 7.0 ± 2.0 in the LigPrep module. Then, all the ligands were redocked into the corresponding complex structures with restriction of all heavy atoms to the reference position. And the interactive free energy contributions of residues within 10 Å of the mass centroids of the native ligands were derived out and analyzed. The experimental values of affinities for PARP-1 ligands were estimated by the referring IC50 values.

According to the free energy contributions and electrostatic properties, residues at the active binding site of PARP-1 were further clustered into three groups, namely electrostatic group, non-electrostatic group and other group. The total free energy contributions of residues in three groups were summed as energy terms of Eele, Enonele, Eother for complex structures respectively. Then, a multiple linear regression model was applied to fit these energy terms to the experimental affinities of the corresponding ligand. The binding affinities (y) was depicted as follows:

$$y=c_{1}\times \;E_{ele}+c_{2}\times \;E_{nonele}+c_{3}\times \;E_{other}+c_{0}$$

### 2.2 Evaluation of molecular docking

The crystal structure in complex with Niraparib (PDB ID: 4R6E) was selected as the receptor for the docking-based virtual screening process. To evaluate the screening power of the receptor, a compound dataset containing both actives and decoys was constructed. 40 ligands of PARP-1 were extracted from the complex crystal structures as the actives. The decoys with structural similarity to actives were generated with an active-to-decoy ratio of 1:50 in the DUD-E server (Mysinger et al., 2012) (http://dude.docking.org/generate) as shown in Supplementary Table S2, and a total of 1990 decoys were generated by deleting the duplicates. Firstly, the docking ability of the Glide module was evaluated by redocking the native ligands (Niraparib) into its binding site using HTVS, SP and XP protocol respectively, and the binding poses with the best docking score were selected for conformational superposition to the original crystal structures. The root mean square deviation (RMSD) was calculated focusing on all heavy atoms of ligands. In order to further evaluate the virtual screening (VS) performance of the receptor (Empereur-Mot et al., 2015), the receiver operating characteristics (ROC) curve with the calculation of area under curve (AUC) was applied to evaluate the discrimination capability between actives and decoys for different docking protocols (Florkowski, 2008).

### 2.3 The workflow of virtual screening and the purchase of selected compounds

The Chemdiv database (https://www.chemdiv.com/) that contains over 1.5 million compounds was applied for the screening of PARP-1 inhibitors. All the compounds were prepared using MMFFs force field, with a target pH of 7.0 ± 2.0, in the LigPrep module. The pocket grid was generated from the crystal structure (PDB ID: 4R6E) and applied in the Virtual Screening Workflow module. All the compounds were firstly prefiltered by Linpinski’s Rules and step-by-step filtered by the docking protocol of high throughput virtual screening (HTVS), standard precision (SP), and extra precision (XP) protocol. The retaining ratios of HTVS, SP, and XP protocol were set as 10%, 10%, and 20% respectively. The top-ranked 2,000 compounds with the best XP docking scores were finally applied for further affinity evaluation by the above multiple linear regression model involved in energy terms of Eele, Enonele, Eother. In order to select the diverse scaffold types, the top 500 compounds with the best predicted affinities were clustered by the *k*-means clustering in Canvas. The representative compounds were picked out for subsequent novelty check in the SciFinder Scholar (https://scifinder.cas.org/). Finally, 20 compounds with proper binding modes and unknown reports of PAPR1 inhibitory activities were selected and purchased from the Taosu Bio-Technique Co., Ltd. (Shanghai, China).

### 2.4 PARP-1 enzyme assays

The ability of 20 selected compounds to inhibit PARP-1 enzyme activity was assessed using Trevigen’s PARP-1 assay kit (Trevigen, cat. No. 4676-096-K) following the manufacturer’s instruction. For PARP inhibitor determination, enzyme assays were conducted in 96-wells FlashPlate (PerkinElmer) with 0.5 U/μl of PARP-1 enzyme, 0.5× activated DNA, 0.5× PARP Cocktail, in a final volume of 50 μl by 1× PARP Buffer. Reactions were initiated by adding NAD+ to the PARP reaction mixture with or without inhibitors and incubated for 60 min at room temperature. 50 μl of 1× Strep-HRP was added to each well to quench the reaction. The plate was sealed and shaken for a further 60 min. Finally, PeroxyGlowTM A and PeroxyGlowTM B were equal volumes and added 100 μl per well. Immediately take chemiluminescent readings by Synergy TM HT (Bio Tek, United States).

### 2.5 The binding mode and energy analysis by molecular dynamics simulation

The initial binding modes of four hit compounds were predicted by docking protocol of XP. A total of four complex conformations were applied in the molecular dynamics (MD) simulation. The geometry optimization and partial charges calculation of hit compounds were performed in Gaussian09 program using HF/6–31G* basis set. The restrained electrostatic potentials (RESP) were assigned using the general AMBER force field (GAFF) (Bhadra and Siu, 2019). Then, all four complex systems were neutralized with sodium ions or chloride ions and immersed in a rectangular TIP3P water box at least 10 Å away from the proteins. All complex systems were parameterized using ff14SB force field (Maier et al., 2015) and performed all-atom molecular simulations in AMBER14 package (Case et al., 2014). The molecular dynamics simulation process was performed in four steps. Firstly, the initial structures were minimized by 2,500 cycles of steepest descent and 2,500 cycles of conjugate gradient. Secondly, the temperature for each system was gradually upgraded from 0 K to 300 K within a period of 100 ps Thirdly, all the heavy atoms of protein and compound were equilibrated with gradually decreasing restraining force constants from 2.0, to 1.5, to 1.0, to 0.5, to 0.1, to 0 kcal mol-1 Å-2 and the simulation time was 100 ps for each restraining force constant. Finally, the molecular dynamics simulations were performed with all the restraints released in the isothermal isobaric (NPT) ensemble with a temperature of 300 K and a pressure of 1 atm. During the simulation, particle mesh Ewald (PME) was used to compute electrostatics in periodic boundary condition and the bonds involving hydrogen atoms were constrained using the SHAKE algorithm. The time step was set as 2 fs, and the trajectories for each system was generated with a production time of 100 ns The MM/GBSA method were further performed to evaluate the binding free energy between the receptor and ligand. 200 snapshots were extracted from the last 20 ns trajectories and used for MM/GBSA calculation, and the parameter settings were referred to the previous works published by our group (Shi et al., 2018; Shi et al., 2019). Then, the per-residue free energy decomposition and root mean square fluctuation were calculated to evaluate the dynamics effect of hit compounds on the binding site of PARP-1 in the complex systems.

### 2.6 Cell proliferation inhibition assay

The human colorectal cancer cells line HCT-116, RKO were obtained from ATCC and cultured in Dulbecco’s modified Eagle medium (DMEM) containing 10% fetal bovine serum (FBS, Gibio), 100 U/ml penicillin (Hyclone) and 100 μg/ml streptomycin (Hyclone). Both cell lines were grown in a humidified atmosphere with 5% CO_2_/95% air at 37°C. The cytotoxic effects of PARP-1 inhibitors were measured by CCK-8. Cells in the logarithmic growth phase were plated in 96-well culture plates. After treatment with Niraparib, compound 8018-7168 and 8018-6529 at indicated concentrations. CCK-8 was added to each well for 4 h at 37°C. Then the optical density value was detected at 450 nm using a microplate reader (Bio Tek). The inhibition rate was calculated from the following equation:

$$Inhibition\;ratio=\left(1-\frac{{OD}_{compound}}{{OD}_{control}}\right)\times 100\%$$

### 2.7 Western blot analysis

After treatment with the corresponding IC50 concentration of Niraparib, compound 8018-6529, 8018-7168 for 48 h, HCT-116 cells were subjected to protein extracted extraction and equivalent amounts of the extraction were separated by SDS-PAGE and transferred onto PVDF membranes. Following blockage of nonspecific sites with 5% skimmed milk powder in TBST, the membranes were incubated with primary antibodies and subsequently subjected to secondary antibodies. ImageJ Software was used to quantify the resulting bands.

### 2.8 Transmission electron microscopy analysis

HCT-116 cells were treated with Niraparib, compound 8018-6529, 8018-7168 at the corresponding IC50 concentration for 48 h. Then the samples were harvested and processed under the instructions. A transmission electron microscope (TEM, JEM-2100) was used to detect the sliced samples. Micrographs were obtained at the magnification of ×25000, *n* = 3.

## 3 Results

### 3.1 The dynamic and energetic characteristics of PARP-1 complex

**FIGURE 1:**
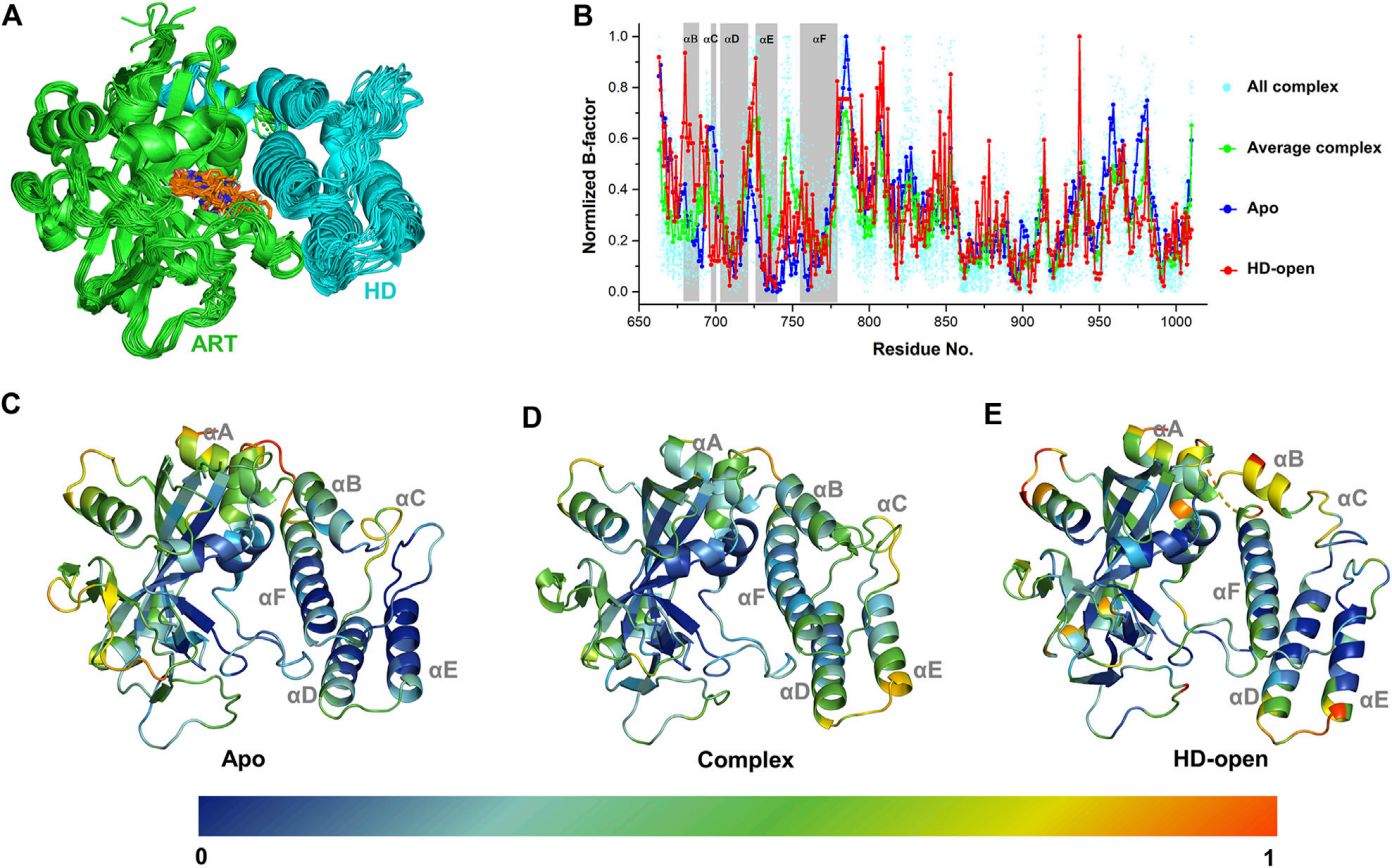
The dynamic characteristics of different conformational states of PARP-1 by B-factor analysis. **(A)** Superposition of 33 PARP-1 complex crystal structures with the existence of ART domain (green) and HD (cyan). The co-crystalized ligands are overlapped in orange sticks. **(B)** The normalized B-factors of apo, complex, HD-open states of PARP-1. The normalized B-factors of 33 complex structures (cyan dot) were averaged and shown in green line. **(C)** The conformational flexibility of apo state, represented by the crystal structure (PDB ID: 4DQY). **(D)** The conformational flexibility of complex state, represented by the crystal structure (PDB ID: 4R6E). **(E)** The conformational flexibility of HD-open state, represented by the crystal structure (PDB ID: 7S6M).

A complete catalytic domain for PARP-1 is consisted of the ART domain and the helix domain (HD). Recently, the HD-open state has been captured in the crystal structures, which shows an active conformation that could prolong interactions with the DNA damage and accounts for allosteric mechanism of PARP-1 (Rouleau-Turcotte et al., 2022). In order to analyze the ligand’s effect on the dynamics and energetic characteristics of the catalytic domain, 33 PARP-1 complex structures with the existence of the HD were selected and analyzed. As shown in Figure 1A, it can be seen that the location superposition of all ligands with diverse binding poses forms a good occupation at the binding site between the ART domain and HD, despite of the minor conformational fluctuation of protein. Different complex structures showed an obvious fluctuation in the distribution of B-factors, and the average value was calculated to reflect the general characteristics of ligand binding (Figure 1B). The dynamics characteristics were further compared between the apo, complex and HD-open states using the normalized B-factors. It can be seen that the main dynamics difference among these states occurs at the HD, and the apo state turns to be more stable comparing to the complex and HD-open states. The normalized B-factors were further projected to different helices of the HD as shown in Figures 1C–E. The αD, αE, αF of HD turn out to be more dynamics in complex state than apo state, suggesting that the binding of ligands could change the conformational flexibility of HD. As a conformational state that binds to DNA damage, the HD-open state shows a conformational shift of HD and a more flexible αA and αB. The conformational association between αA/αB and αF seems to be the main switch for HD-open state and the interaction with αF could be a useful approach to intervene allosteric effect of PARP-1.

**FIGURE 2:**
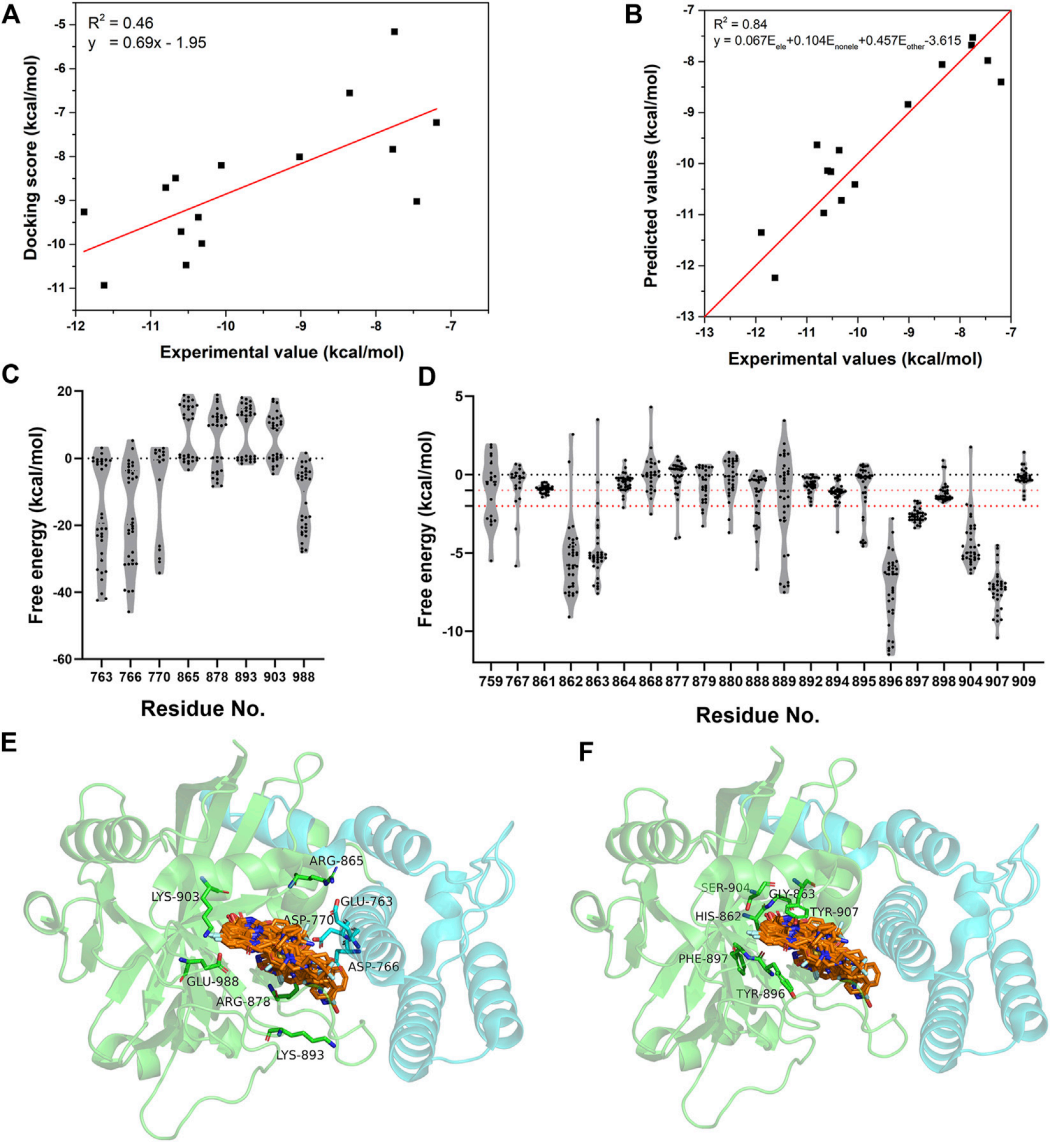
The energetic characteristics of PARP-1 complex by per-residue energy decomposition in molecular docking. **(A)** The linear relationship between the docking scores and experimental activities for 15 PARP-1 ligands. **(B)** The linear relationship between the predicted activities and experimental activities by a multiple linear regression model. **(C,E)** The per-residue energy distribution and location of electrostatic residue group on PARP-1. **(D,F)** The per-residue energy distribution and location of non-electrostatic residue group on PARP-1.

The energetic characteristics of PARP-1 complex were further analyzed by molecular docking. All ligands were redocked with a conformational constraint to the native poses and the corresponding docking scores were applied for binding free energy analysis. As the experimental activities for some ligands were missing or inconsistent among different reports, the ligands were applied only if their activities exist at the same level of magnitude in different reports and the experimental values were depicted by the average of IC50 values. As shown in Figure 2A, a total of 15 ligands with valid activities showed linear correlation with their docking scores with R2 of 0.46. The per-residue free energy decomposition was further performed for all complex structures, and the energy contributions of the pocket residues were evaluated by the distribution probabilities of values. As shown in Figures 2C,E, eight electrostatic residues show obvious energetic perturbation for the binding free energies of PARP-1 ligands. The negative-charged residues including Glu763, Asp766, Asp770, and Glu988 have positive effect for binding, while the positive-charged residues including Arg865, Arg878, Lys893, and Lys903 have negative effect. This electrostatic interaction preference may account for the fact that most ligands of PARP-1 are positive-charged. It can be seen that Glu763, Asp766, Asp770 are all located on αF of HD, suggesting the electrostatic attraction between the ligands and these residues could affect the interaction and stability of HD. The other non-electrostatic residues also show great effect on the binding free energies (Figures 2D,F). It can be seen that His862, Gly863, Tyr896, Phe897, Ser904, Tyr907 have key interactions for almost all complex structures, while other hydrophobic or polar residues at the binding site show different interactions among different ligands. According to the electrostatic property, the residues involved in the ligand binding were classified into three groups, namely the electrostatic group, the non-electrostatic group and the others. To evaluate the effect of different residue groups, the energy contribution of each group was calculated as shown in Table 1. A multiple linear regression model was built with R2 of 0.84 as follows: $y=0.067\;E_{ele}+0.104\;E_{nonele}+0.457\;E_{other}-3.615$ , and the predicted activities showed a good consistency with the experimental activities (Figure 2B). It is suggested that the residue classification and multiple linear regression are useful approach to improve the accuracy of predicted values by molecular docking method, and could be further applied for the affinity predicted during the virtual screening.

**TABLE 1:** The calculated energy contribution of three residue groups and the predicted activities by a multiple linear regression model.

PDB	Eele^a^	Enonele^b^	Eother^c^	Predicted^d^	Experimental^e^
1UK0	−27.60	−31.85	−1.51	−9.47	−10.80
2RD6	−27.05	−37.99	0.12	−9.33	—
3GN7	−21.43	−38.54	−1.28	−9.64	—
3L3M	−16.65	−48.90	0.07	−9.78	—
4GV7	−3.40	−32.29	−1.71	−7.98	−7.46
4HHY	−24.67	−45.16	−3.04	−11.35	−11.89
4HHZ	−11.12	−42.76	−2.97	−10.16	−10.53
4L6S	−56.25	−40.28	−1.47	−12.24	−11.62
4OPX	−7.78	−27.32	−1.53	−7.68	−7.77
4OQA	−1.74	−30.47	−2.54	−8.06	−8.35
4OQB	−8.30	−21.89	−2.37	−7.53	−7.75
4R5W	−8.32	−43.07	−1.75	−9.45	—
4R6E	−33.44	−50.87	0.08	−11.11	—
4RV6	−36.45	−38.64	0.62	−9.79	—
4UND	−8.50	−37.30	−1.67	−8.83	—
4UXB	−15.81	−37.64	4.25	−6.65	—
4ZZZ	−3.87	−35.54	−1.81	−8.40	−7.19
5A00	−32.98	−45.02	1.68	−9.74	−10.37
5HA9	−25.28	−20.94	−2.15	−8.47	—
5KPN	−21.31	−51.57	−1.94	−11.29	—
5KPO	−20.21	−52.82	−1.96	−11.36	—
5KPP	−15.33	−54.20	−1.79	−11.10	—
5KPQ	−12.84	−50.57	−2.14	−10.71	—
5WRQ	−52.56	−47.39	−2.40	−13.16	—
5WRY	−49.05	−54.66	−1.85	−13.43	—
5WRZ	−31.97	−34.18	1.15	−8.78	—
5WS0	−37.67	−38.08	−0.68	−10.41	−10.06
5WS1	−35.74	−39.50	−1.86	−10.97	−10.67
5WTC	−16.65	−52.36	−1.63	−10.92	—
5XSR	−39.98	−43.86	1.56	−10.14	−10.59
5XST	−40.52	−51.08	2.02	−10.72	−10.32
5XSU	−32.63	−40.62	2.60	−8.84	−9.02
6GHK	−4.84	−50.47	−1.82	−10.02	—

^a^ The sum of the residue energy terms for residues in the electrostatic group.

^b^ The sum of the residue energy terms for residues in the non-electrostatic group.

^c^ The sum of the residue energy terms for the other residues except the electrostatic group and non-electrostatic group.

^d^ The predicted binding free energies calculated by the multiple-linear regression model for the native ligand in the corresponding complex.

^e^ The experimental binding free energies calculated by the average IC50 according to the following equation: $∆G=-RT\ln\left(1/{IC}_{50}\right)$ .

### 3.2 The virtual screening workflow and discovery of new hit compounds

**FIGURE 3:**
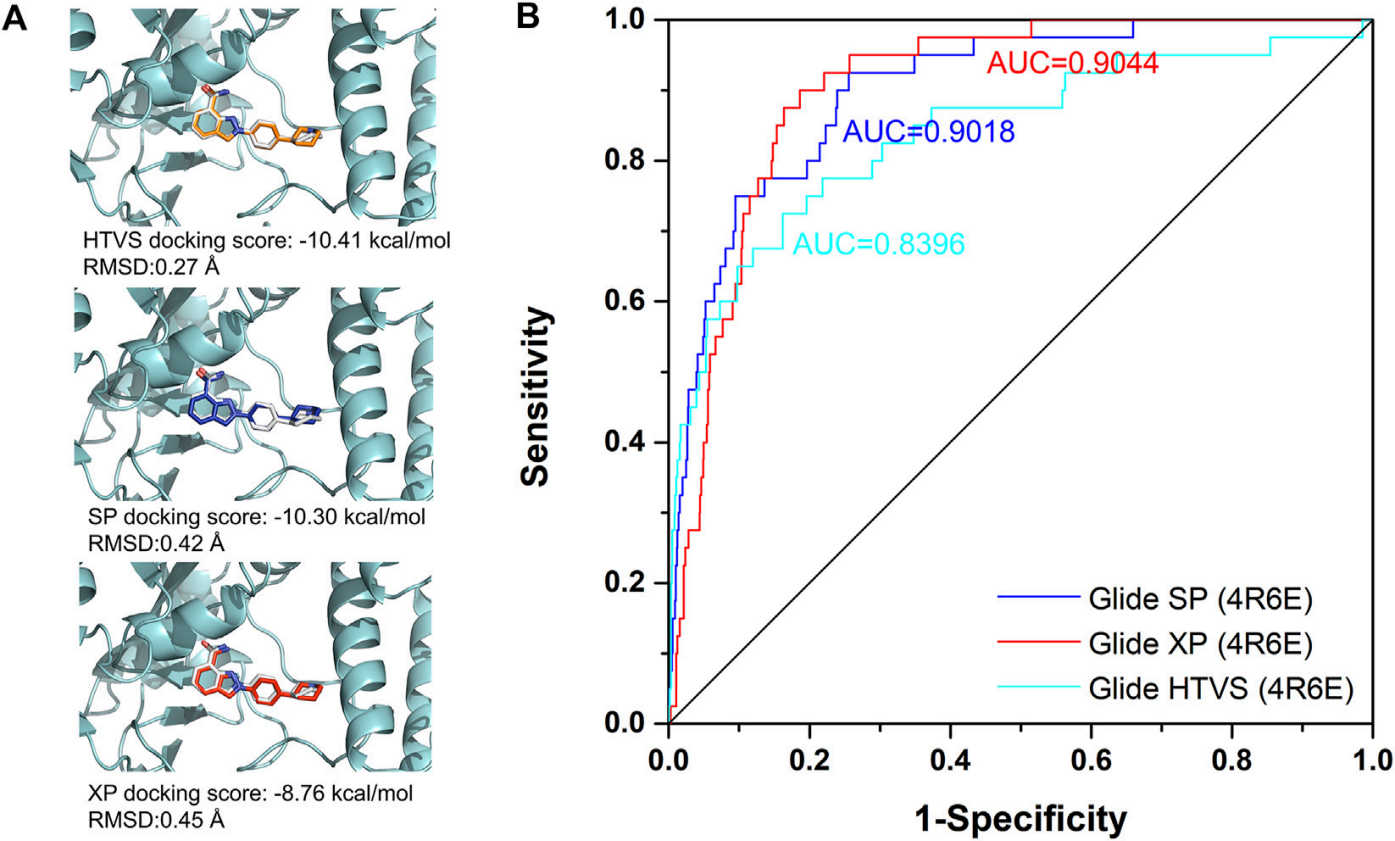
The evaluation of docking power and screening power for crystal structure in complex with Niraparib (PDB ID: 4R6E). **(A)** The evaluation of the docking power of crystal structure of 4R6E by HTVS, SP, and XP protocol respectively. **(B)** The screening power of the crystal structure of 4R6E by HTVS, SP, and XP protocol.

Among all the complex states, the crystal structure in complex with Niraparib (PDB ID: 4R6E) was selected for the docking-based virtual screening. The docking power of different docking protocols (HTVS, SP, XP) in Schrödinger 2015 were firstly evaluated by redocking the native ligands into the original crystal structures. As shown in Figure 3A, Niraparib were redocked into the pocket of PARP-1 with a good overlapping to the native pose with RMSD lower than 2 Å, indicating that the native pose could be successfully predicted by different docking protocols. Then, the screening power of different docking protocols were further tested. A dataset of 2030 compounds (including 40 actives and 1990 decoys) were built and applied to perform molecular docking. The screening power was evaluated by area under curve (AUC) of the receiver operating characteristics (ROC) curve. As shown in Figure 3B, the AUC values for HTVS, SP, XP were 0.8396, 0.9018, 0.9044 respectively and large enough for the docking-based virtual screening process. Specially, with the improvement of docking accuracy from HTVS to SP to XP, there is an increasing trend in screening capacity. Therefore, the docking protocols with crystal structure of 4R6E for was suitable for the following docking-based virtual screening workflow.

**FIGURE 4:**
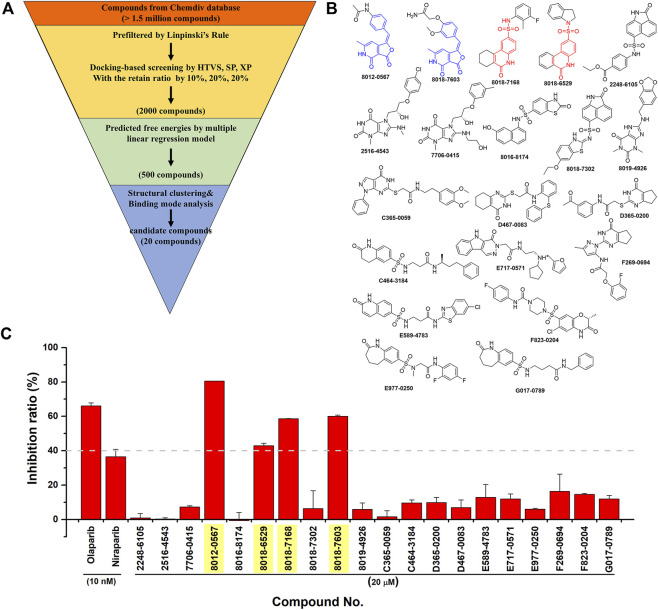
The integrated *in silico* screening workflow and *in vitro* inhibitory activity assay of candidate compounds. **(A)** The *in silico* screening workflow. **(B)** The chemical formula of 20 compounds selected for *in vitro* assay. The scaffolds of four hit compounds are labeled out with arylidenefuropyridinediones (compound 8012-0567 and 8018-7603) in blue and 1,2-Dihydro-2-oxo-6-quinolinesulfonamide (compound 8018-7168 and 8018-6529) in red. **(C)** The inhibitory activities of two positive controls and 20 candidate compounds.

Then, an integrated *in silico* screening workflow was performed to get the candidate compounds targeting PARP-1. As shown in Figure 4A, over 1.5 million compounds in Chemdiv database were firstly pre-filtered by the Linpinski’s Rules, and the left compounds were successively screened by the docking protocols of HTVS, SP and XP with the retain ratio of top 10%, 20%, 20% respectively. A total 2,000 compounds were selected with the top-ranking XP docking scores. A per-residue free energy evaluation was further performed to predict the binding affinity of those compounds, and the top 500 compounds were retained. In order to ensure as much structural diversity as possible with the fewest compounds, the structural clustering was performed and 20 candidate compounds were finally selected and purchased for *in vitro* assay (Figure 4B). The inhibitory activities *in vitro* were performed using Trevigen’s PARP-1 assay kit. As shown in Figure 4C, the inhibition ratios of two positive controls (Olaparib and Niraparib) were firstly tested under 10 nM with values of 66.1% and 36.7%, which were consistent with the corresponding range of IC50 activities. Then, the inhibition ratios of all candidate compounds were evaluated under 20 μM, and a cutoff value of 40% were set to select the potential hit compounds. Four compounds (Chemdiv codes: 8012-0567, 8018-6529, 8018-7168, 8018-7603) showed obvious inhibitory activities against PARP-1 than other compounds with values of 80.5%, 42.9%, 58.7%, 60.0% respectively. The corresponding chemical formulas can be found in Figure 4B. It can be seen that 8012-0567 and 8018-7603 have the same scaffold unit of arylidenefuropyridinediones, while 8018-6529 and 8018-7168 have the same structural fragments of 1,2-Dihydro-2-oxo-6-quinolinesulfonamide. All these hit compounds have no report of PARP-1 inhibition activity before.

### 3.3 The energetic and dynamic effect of hit compounds on PARP-1

The binding free energies of the equilibrium complex states for four hit compounds were evaluated by the conformational sampling of last 20 ns trajectories of molecular dynamics simulation and further calculated by the MM/GBSA method as shown in Table 2. It can be found that the calculated binding free energies (ΔH) have a good consistency with the experimental inhibition ratio for 8012-0567/8018-7603 and 8012-6529/8018-7168. The ΔH was then split into polar parts (ΔEele, ΔEele,sol) and non-polar parts (ΔEvdw, ΔEnonpl,sol). For all the four hit compound systems, the energy terms ΔEele, ΔEvdw, ΔEnonpl, sol were favorable for ligand binding while the polar interaction contribution by solvent (ΔEele,sol) was adverse. The electrostatic interaction (ΔEele) was much stronger in 8012-0567 complex than 8018-7603 complex, which mainly accounts for the free energy difference between these two compounds. As for 8012-6529/8018-7168 complex systems, the van der Waals interaction (ΔEvdw) and electrostatic interaction by solvent (ΔEele,sol) in 8018-7168 system were both optimized than 8012-6529.

**TABLE 2:** The binding free energies of four hit compounds evaluated by MM/GBSA.

Terms (kcal/mol)	8012-0567	8018-7603	Δ^a^	8018-7168	8018-6529	Δ^b^
ΔEele^c^	−48.00 ± 5.58	−41.18 ± 10.34	−6.82 ± 10.75	−25.15 ± 5.51	−24.84 ± 4.16	−0.31 ± 6.90
ΔEvdw^d^	−43.96 ± 2.53	−43.62 ± 2.95	−0.34 ± 3.89	−46.63 ± 2.98	−44.75 ± 2.67	−1.88 ± 4.00
ΔEele,sol^e^	54.42 ± 3.89	54.53 ± 7.34	−0.11 ± 8.31	38.43 ± 3.74	40.04 ± 2.97	−1.61 ± 4.78
ΔEnonpl,sol^f^	−5.33 ± 0.11	−5.69 ± 0.18	0.36 ± 0.21	−6.08 ± 0.14	−5.82 ± 0.12	−0.26 ± 0.18
ΔH^g^	−42.88 ± 2.84	−35.96 ± 3.68	−6.92 ± 4.65	−39.44 ± 2.93	−35.36 ± 2.65	−4.08 ± 3.95
Inhibition ratio	80.5 ± 0.1%	60.0 ± 0.8%		58.7 ± 0.1%	42.9 ± 1.4%	

^a^ The energy difference between compound 8012-0567 and 8018-7603.

^b^ The energy difference between compound 8018-7168 and 8018-6529.

^c^ The electrostatic energy term.

^d^ The Van der Waals energy term.

^e^ The polar solvation free energy term.

^f^ The non-polar solvation free energy term.

^g^ The total binding free energy as the sum of ΔEele, ΔEvdw, ΔEele, sol, ΔEnonpl,sol.

**FIGURE 5:**
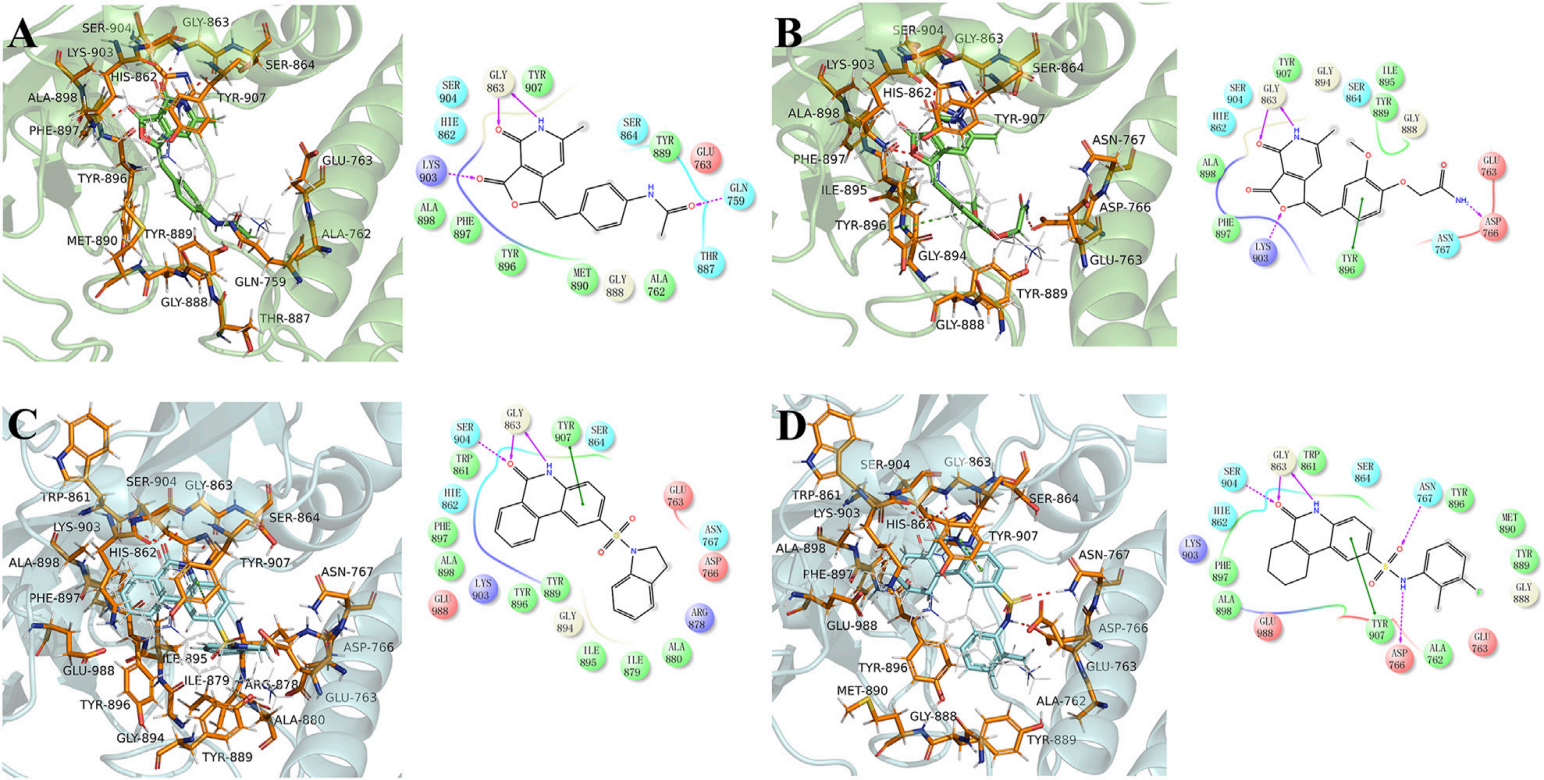
The binding mode analysis of four hit compounds, 8012-0567 **(A)**, 8018-7603 **(B)**, 8018-6529 **(C)**, 8018-7168 **(D)** in the equilibrium complex states. The equilibrium conformation for each hit compound was represented by the last snapshot during the 100 ns simulation trajectory. The PARP-1 was shown in green or cyan cartoon, while the pocket residues within 4 Å of the ligand atoms were shown in orange sticks. The ligands were shown in cyan/green sticks, and Niraparib was shown in gray lines for location comparison. The hydrogen binding and π-π stacking interactions were shown red and green lines in 2D and 3D interaction plots respectively.

**FIGURE 6:**
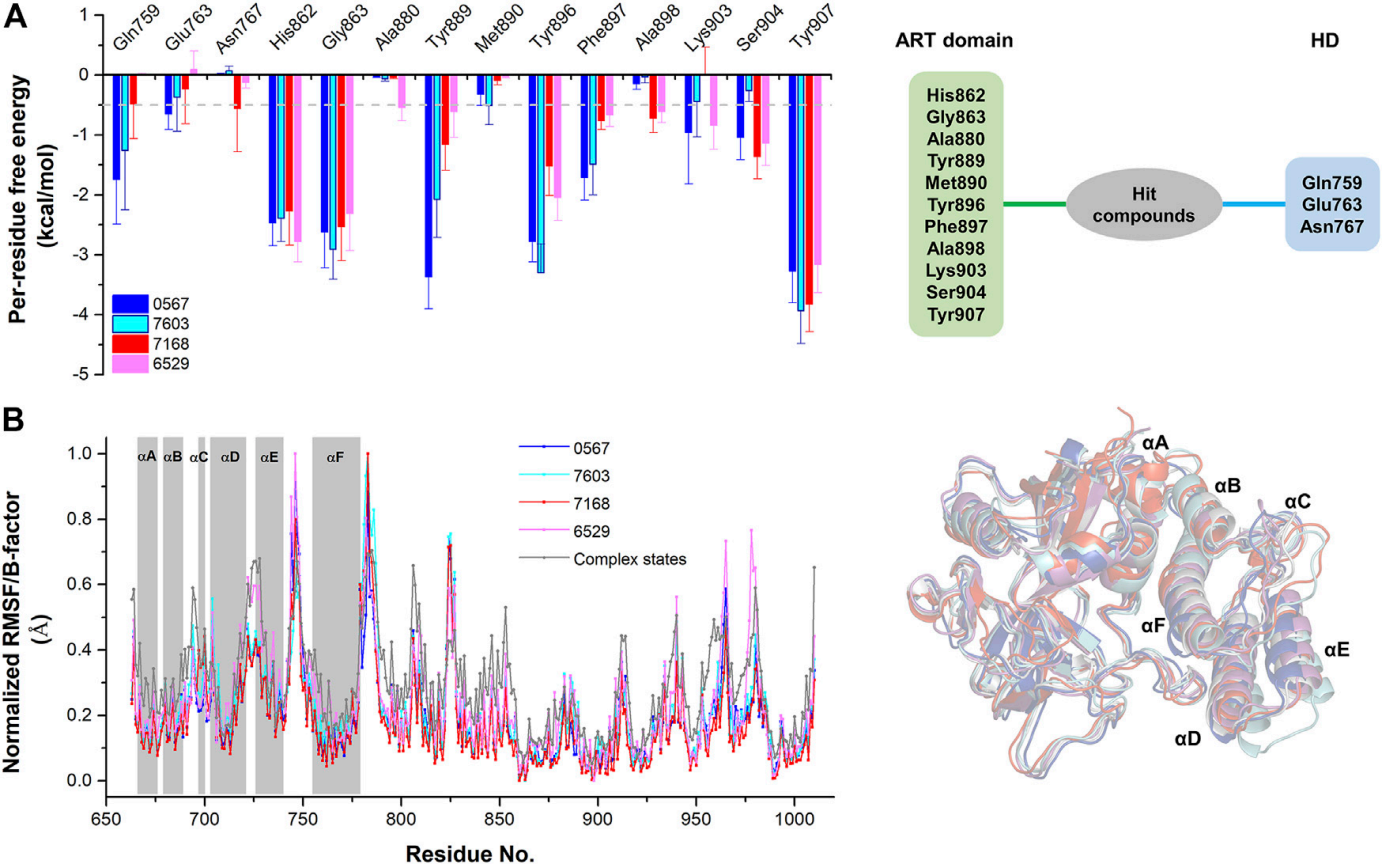
The per-residue free energy decomposition and conformational analysis of the equilibrium conformations of four hit compounds by molecular dynamics. **(A)** The per-residue free energy decomposition of the key residues on ART domain and HD. **(B)** The normalized RMSF analysis of the equilibrium conformations of four hit compounds compared to the average B-factors of the complex states. The equilibrium conformations of four hit compounds were superposed with the crystal structures (PDB ID: 4R6E).

The equilibrium complex states for four hit compounds were achieved from the last snapshot in the molecular dynamics. The binding modes were further characterized by the interactions between ligands and the adjacent residues as shown in Figure 5. Compounds 8012-0567 and 8018-7603 have similar location superposition with the binding pose of Niraparib. The scaffold of 8012-0567/8018-7603 forms three hydrogen bonds with the backbone atoms of Gly863 and the sidechain of Lys903 in the ART domain. The different substituted groups on the benzene ring affect the minor conformational shift of the ring and different interactions with the residues in αF of HD. 8012-0567 has a hydrogen bonding with Gln759, while 8018-7603 has π-π stacking with Tyr896 and hydrogen bonding with Asp766 (Figures 5A,B). However, compounds 8012-6529 and 8018-7168 have quite different location superposition comparing to binding pose of Niraparib. The scaffold of 8012-6529/8018-7168 also form three hydrogen bonds with the backbone atoms of Gly863 and the sidechain of Ser904, and a π-π stacking interaction with the scaffold benzene ring in the ART domain. The binding poses of 8012-0567/8018-7603 are also affected by the substituted groups. The sulfonamide group of 8012-7168 forms hydrogen bonds with Asp766 and Asn767 in αF of HD, while sulfonamide group of 8018-6529 forms no hydrogen bonds. (Figures 5C,D). The further per-residue energy decomposition in Figure 6A shows that His862, Gly863, Ala880, Tyr889, Met890, Tyr896, Phe897, Ala898, Lys903, Ser904, Tyr907 in ART domain and Gln759, Glu763, Asn767 in HD have the significant energy contribution for the binding of four hit compounds. Consistent with the known inhibitors, His862, Gly863, Tyr889, Tyr896, Phe897, Tyr907 act as the core residues with the main energy contributions for all hit compounds, while other residues provide different energy contribution based on the difference of substitutions. When interacting with the HD, Gln759 and Glu763 have obvious contribution for the binding of 8012-0567/8018-7603, while Gln759 and Asn767 benefit for the binding of 8012-7168. The dynamic effect of the equilibrium complex states induced by hit compounds were further characterized by the normalized root mean square fluctuation (RMSF). The equilibrium complex state predicted by molecular dynamics has the similar dynamic profile with the complex crystal structures as shown in Figure 6B. It can be seen that the main conformational fluctuations occur at the αD-αE loop and αE-αF loop of the HD upon the ligand binding, which shows obvious conformational shift among the equilibrium complex states.

### 3.4 The anti-tumor activity evaluation and autophagy mechanism

**FIGURE 7:**
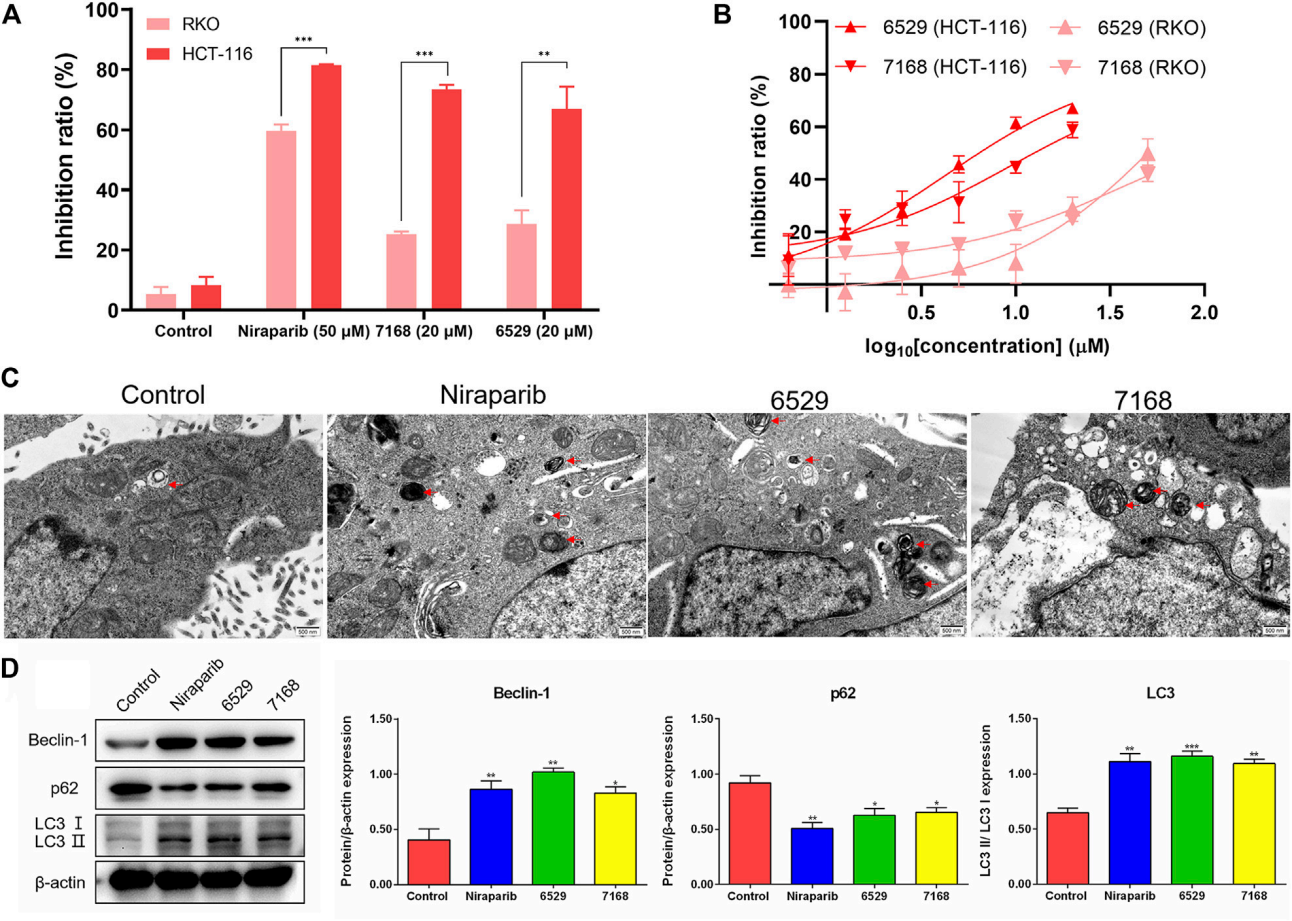
Discovered hit compounds inhibit cell survival and induced the autophagy in a human colorectal carcinoma cell line HCT-116. **(A)** The inhibition ratio of two hit compounds (8012-0567, 8018-7603) at 20 μM against the cell survival of human colorectal carcinoma cell lines (HCT-116, RKO), Niraparib at 50 μM as the positive control. Cell viability was determined by the CCK-8 assay (*n* = 3). (*p < 0.05, **p < 0.01, ***p < 0.001) **(B)** the human colorectal carcinoma cell lines (HCT-116, RKO) were treated with different concentrations of 8018-6529 and 8018-7168. The IC50 values were predicted by utilizing the GraphPad^®^ program. **(C)** TEM detection of autophagosomes accumulation in HCT-116 cells treated with compound 8018-6529 and 8018-7168 for 24 h, Niraparib as the positive control. **(D)** Western blot analysis was performed after treatment of compound 8018-6529 and 8018-7168 to demonstrate the expression of Beclin-1, p62, and LC3 II/I protein, markers for autophagy, Niraparib as the positive control. Densitometric values of Beclin-1, p62 and LC3 compared with their respective total proteins were evaluated *via* ImageJ software and are presented as the mean ± SD (*n* = 3) (*p < 0.05, **p < 0.01, ***p < 0.001).

To validate the anti-tumor activities of these hit compounds of PARP-1, HCT-116 (BRCA-deficient colorectal carcinoma cells), RKO (BRCA-proficient colorectal carcinoma cells) were applied for the cell survival assay. As shown in Figure 7A, compounds 8018-7168 and 8018-6529 out of the four hit compounds have significant inhibition effect against the cell growth for both cell lines at 20 μM. Considering the synthetic lethality of PARP-1 inhibitors against BRCA-deficient cells, it can be seen that the inhibition ratio of these two compounds is significantly higher in HCT-116 than RKO in the colorectal carcinoma cell lines, which is also consistent with the results of Niraparib. Specially, compounds 8018-7168 and 8018-6529 show obvious anti-tumor activities against HCT-116 cell lines and the IC50 were further tested as 9.29 μM and 4.30 μM respectively, which showed better inhibitory effect than Niraparib (Figure 7B; Table 3; Supplementary Figure S1). The previous studies demonstrated that autophagy was initiated in a series of cancer cell lines after the treatment of PARP-1 inhibitors, but no report for HCT-116 cell lines. To further demonstrate the induction of autophagy, we firstly investigated the ultrastructure by transmission electron microscopy (TEM) before and after the treatment of compounds 8018-7168 and 8018-6529. TEM images clearly demonstrate that these two compounds could induce the process of autophagy by generating more autophagosomes compared with untreated cells (Figure 7C). Then, the level of three autophagy-related proteins, Beclin 1, sequestosome 1 (SQSTM1/p62) and microtubule-associated protein 1 light chain3 (LC3) were detected in HCT-116 with the treatment of compounds 8018-7168 and 8018-6529 (Niraparib for positive control) by immunoblot analysis. Figure 7D showed that compounds 8018-7168 and 8018-6529 obviously increased the level of Beclin 1 and LC3 II/I, and reduced the level of p62 in HCT-116 cells, which further proved the induction of autophagy.

**TABLE 3:** The IC50 values of anti-tumor activities of compound 8018-7168, 8018-6529 and Niraparib against HCT-116 and RKO cell lines. The unit of concentration is μM.

Cell lines	8018-7168	8018-6529	Niraparib
HCT116	4.30	9.30	33.09
RKO	73.27	35.03	40.89

## 4 Discussion

The clinical efficacy of small molecule compounds is affected by a series of factors and mechanisms, which is even pronounced for the targeted inhibitors of PARP-1 (Rose et al., 2020). In addition to competitive binding to the substrate binding site of NAD+, some PARP-1 inhibitors can also affect the conformational state of PARP-1 through allosteric regulation mechanism, thereby affecting its retention time binding to the damaged DNA (Zandarashvili et al., 2020). It has been confirmed that the allosteric effect has a close and direct relationship with the helix domain (HD), and small-molecule inhibitors can affect the conformational state and dynamic characteristics of HD through the interaction with HD residues (Rouleau-Turcotte et al., 2022). In this study, we tried to analyze energetic and dynamic effect of reported small-molecule inhibitors on the HD, so as to reveal the allosteric regulatory effect of small-molecule binding. We preliminarily studied the residue energy contribution and B-factor in the complex states by a systematic analysis of crystal structures with the existence of the HD. It is found that the interactions with HD are mainly related to the electrostatic residues including Glu763, Asp766, Asp770 on αF helix of HD, and the mutual interactions might further cause the conformational changes of other helices on HD, thus eventually result in the HD-open state. These results provide the initial hint for the following *in silico* and *in vitro* screening.

In the process of per-residue energy analysis by molecular docking, it was found that there was a better multiple-linear relationship between three energy terms (Eele, Enonele, Eother) and the experimental activities of the reported inhibitors, when comparing to the linear relationship between the docking scores and the experimental activities. These energy terms were achieved by classifying the pocket residues of PARP-1 according to electrostatic properties, and summing the contributions of residues in each group. Meanwhile, we applied this multiple-linear model for *in silico* screening process. Since the high false positive rate is always a big problem in virtual screening (Adeshina et al., 2020; Bender et al., 2021), other strategies were also designed to ensure the reliability of virtual screening during the whole process, including the evaluation of docking and screening abilities, structural diversity analysis and the inspection of binding modes. Trevigen’s PARP-1 assay kit assays confirmed that the *in silico* screening process was quite successful, with a hit ratio of 20%, despite that the inhibitory activities was still at the micromolar level. For the further investigation of active scaffolds of four hit compounds, it was found that the PARP-1 inhibitory activity had never been reported for these compounds before. What’s more, the scaffold unit of arylidenefuropyridinediones for 8012-0567 and 8018-7603 was previously reported to show Topoisomerase 1 (LdTop1) (Mamidala et al., 2016) and α-glucosidase (Bathula et al., 2015) inhibition activities. As PARP-1 has broad synergistic effect with other targets in anti-tumor studies (Chang et al., 2021; Wei et al., 2021), these compounds also provide the alternative active scaffold for the further multi-targeting drug design by combining PARP-1 with LdTop1 or α-glucosidase.

In the course of the binding mode and energy analysis of the hit compounds by molecular dynamics, we found that four compounds have strong interaction with key residues including His862, Gly863, Tyr889, Tyr896, Phe897, Tyr907, which is consistent with the known inhibitors (Jagtap and Szabo, 2005; Curtin and Szabo, 2020). It was also noticed that these compounds could form hydrogen bonding with residues like Gln759, Asp766, Asn767 on the HD, which was also shown correspondingly in the energy analysis. The effect of these hit compounds on HD was not quite significant from the energetic aspect, and the overall dynamic characteristics were similar to the conformations of known small-molecule complexes of PARP-1. One possible reason is that the substituent groups of the compounds, especially the structural part interacting with the HD is not big enough to form strong interactions. Therefore, the further structural modification against substituent groups of the compounds may effectively improve the interactions with HD. From the per-residue energy decomposition, it was found that further improving the electrostatic interaction (ΔEele) for arylidenefuropyridinedione of 8012-0567 and 8018-7603 or further improving the van der Waals interaction (ΔEvdw) or electrostatic interaction by solvent (ΔEele,sol) for 1,2-Dihydro-2-oxo-6-quinolinesulfonamide of 8012-6529 and 8018-7168 might be able to optimize the inhibitory activities of the corresponding derivatives.

The cell experiments were further applied to confirm the anti-tumor abilities of the four hit compounds, specially by the colorectal carcinoma cell lines with BRCA deficiency or not. The results showed that two out of four hit compounds had validated inhibitory effect against two human colorectal carcinoma cell lines (HCT-116, RKO), and the better inhibitory effect of these compounds against HCT-116 than RKO was consistent with the results of Niraparib. As the signaling pathways of tumor growth are quite complex and may be regulated by a series of factors, the detailed mechanism of the anti-tumor specificity for two hit compounds for HCT-116 still need further investigation in the future. Compounds 8012-6529 and 8018-7168 showed significant concentration-dependent inhibitory effect against HCT-116, and the IC50 values both reached the micromolar level. Previously, the induced autophagy by PARP-1 inhibitors was reported in other tumor cells (Arun et al., 2015; Liu et al., 2019; Santiago-O'Farrill et al., 2020; Zai et al., 2020), but not in HCT-116. Further western blot analysis and transmission electron microscopy analysis of 8012-6529 and 8018-7168 confirmed the induction of autophagy. It was suggested that the anti-tumor effect of PARP-1 inhibitors could be further enhanced by combination use with autophagy inhibitors. The results of this study also suggested the possibility of 8012-6529 and 8018-7168 for combination use against the killing of HCT-116.

Overall, four hit compounds with obvious inhibitory activities targeting PARP-1 were discovered through *in silico* and *in vitro* screening. Further cell assays showed that compounds 8018-6529 and 8018-7168 could inhibit the growth of the human colorectal cancer cell (HCT-116) with IC50 values of 4.30 and 9.29 μM and were accompanied with induced autophagy process. The suggestions on the structural modification of these compounds were also provided by the binding mode and energy analysis. The results in this study could provide potential hit compounds for the development of anti-cancer drug.

## Data Availability

The original contributions presented in the study are included in the article/Supplementary material, further inquiries can be directed to the corresponding authors.
