# Supplementary material for: Discovery of novel anti-tumor compounds targeting PARP-1 with induction of autophagy through in silico and in vitro screening
Source: Front Pharmacol. 2022 Oct 24;13:1026306. doi: 10.3389/fphar.2022.1026306 (PMC9638114; doi:10.3389/fphar.2022.1026306)
Supplement: Supplementary file 3 [file DataSheet1.docx]

Supplementary Material

## Supplementary Figures


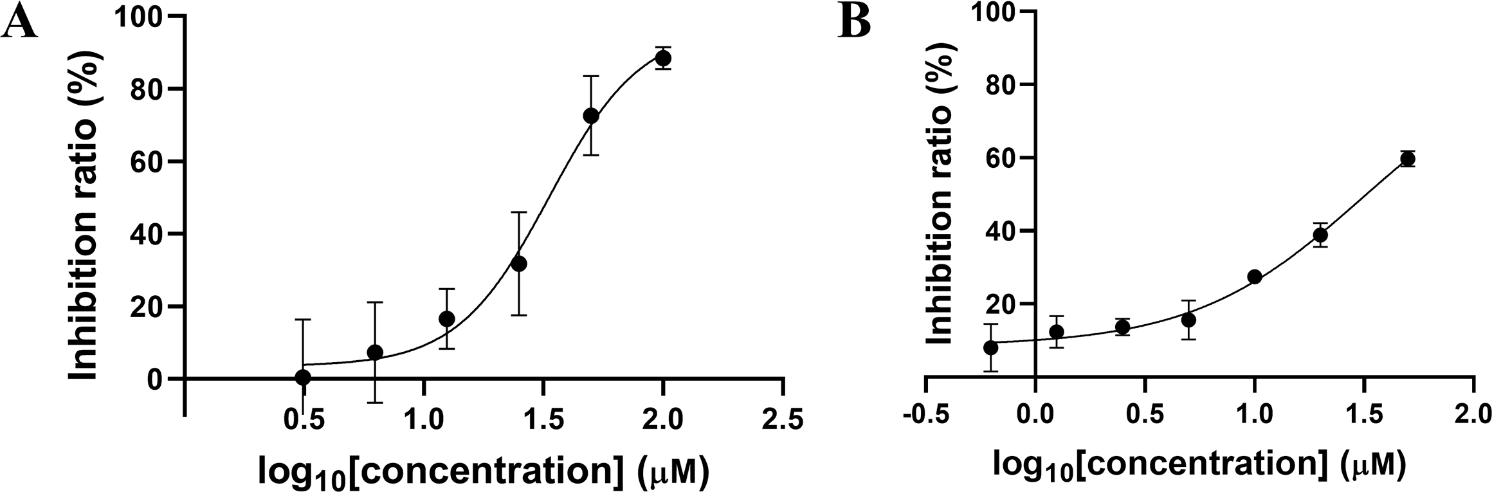


**Supplementary Figure 1.** The human colorectal carcinoma cell lines HCT-116 (A), RKO (B) were treated with different concentrations of Niraparib. The inhibitory activities of IC50 values were predicted by utilizing the GraphPad® program.
